# COMMENTARY ON: EFFECT OF INSPIRATORY MUSCLE TRAINING ON INSPIRATORY MUSCLE STRENGTH IN ADULTS WITH POST-COVID-19 CONDITION AND INSPIRATORY MUSCLE WEAKNESS: A RANDOMIZED CONTROLLED TRIAL

**DOI:** 10.2340/jrm.v58.46254

**Published:** 2026-07-09

**Authors:** Raju KUMAR, Palakdeep KAUR, Urvashi CHAUHAN

**Affiliations:** Department of Physiotherapy, Maharishi Markandeshwar Institute of Physiotherapy and Rehabilitation, Maharishi Markandeshwar (Deemed to be University), Mullana, Ambala, Haryana, India

*To the Editor,*

We read with great interest the randomized controlled trial by Tornberg et al. evaluating inspiratory muscle training (IMT) combined with physical exercise in individuals with post-COVID-19 condition (PCC) and inspiratory muscle weakness (1). The authors should be commended for addressing an important and clinically relevant rehabilitation challenge in a population that continues to experience persistent symptoms long after acute infection. The study contributes valuable evidence by demonstrating that IMT may improve inspiratory muscle strength and cough-related symptoms in individuals with objectively documented inspiratory muscle weakness (1). Given the growing need for effective rehabilitation strategies for PCC, these findings are both timely and clinically meaningful. We would, however, like to discuss the interpretation of these physiological improvements in relation to patient-centred recovery outcomes, as well as the implications of the feasibility challenges encountered during the study.

A notable finding was the significant improvement in maximal inspiratory pressure (MIP) observed in the intervention group compared with the active control group (1). Furthermore, responder analyses suggested that participants receiving IMT were more likely to achieve clinically meaningful improvements in inspiratory muscle strength (1). These findings support the physiological rationale for respiratory muscle training in individuals with PCC and are consistent with previous reports demonstrating benefits of IMT in post-COVID patients (2, 3). Nevertheless, the study demonstrated significant between-group differences primarily for MIP and cough frequency, whereas most patient-centred outcomes, including fatigue severity, dyspnoea, physical activity, and health-related quality of life, did not differ significantly between groups. Although the authors appropriately acknowledge the possibility that the modest sample size limited statistical power, the observed discrepancy between physiological improvement and broader clinical recovery warrants further discussion.

From a rehabilitation perspective, improvements in physiological parameters are important but are rarely considered the ultimate goal of treatment. Rehabilitation programmes are primarily intended to reduce symptom burden, enhance participation, improve functional performance, and support meaningful engagement in everyday activities (4, 5). Consequently, an important question arises regarding the extent to which gains in inspiratory muscle strength translate into outcomes that are directly relevant to patients’ daily lives. While stronger inspiratory muscles may represent a favourable biological adaptation, the absence of corresponding between-group improvements in fatigue, dyspnoea, and quality of life suggests that the relationship between physiological recovery and patient-centred recovery may be more complex than is currently understood. Future investigations might include whether specific thresholds of physiological improvement are required before meaningful functional benefits are seen.

The study also highlights important feasibility considerations. Of the eligible individuals identified during recruitment, many were unable to participate, declined participation because of severe fatigue or post-exertional symptom exacerbation (PESE), or withdrew during the intervention period because of worsening symptoms following physical exertion. These observations are particularly noteworthy because individuals experiencing severe fatigue and PESE frequently represent those with the greatest rehabilitation needs. As a result, the benefits observed in the present trial may primarily reflect outcomes among individuals capable of tolerating structured rehabilitation programmes rather than the broader population of people living with PCC. This issue is not merely methodological; it has direct implications for clinical implementation and the generalizability of rehabilitation interventions in real-world settings.

An especially interesting aspect of the discussion is the authors’ suggestion that IMT may be a feasible strategy for selected individuals with limited tolerance for physical exertion. This observation deserves further consideration. The present study evaluated IMT in combination with exercise, making it difficult to determine whether IMT alone could provide meaningful benefits in individuals who are unable to tolerate conventional exercise-based rehabilitation. Given the challenges associated with PESE (6, 7), it is conceivable that IMT monotherapy may represent a more accessible entry point into rehabilitation for certain subgroups of patients. Future trials specifically investigating IMT as a stand-alone intervention, particularly in individuals with severe PESE, may help clarify whether respiratory muscle training can serve as a bridge towards broader rehabilitation participation or as an alternative strategy for those unable to engage in traditional exercise programmes.

In conclusion, Tornberg et al. have made a significant contribution to the emerging literature on PCC rehabilitation, and provided encouraging evidence in support of a potential role for IMT in individuals with inspiratory muscle weakness (1). We believe their findings also raise broader questions regarding the relationship between physiological improvement and patient-centred recovery, as well as the feasibility of rehabilitation interventions among individuals with greater symptom burden. Future research addressing these issues may help to refine rehabilitation strategies and clarify the role of IMT in individualized care path-ways for people living with PCC.
