# Supplementary material for: COMMENTARY ON: EFFECT OF INSPIRATORY MUSCLE TRAINING ON INSPIRATORY MUSCLE STRENGTH IN ADULTS WITH POST-COVID-19 CONDITION AND INSPIRATORY MUSCLE WEAKNESS: A RANDOMIZED CONTROLLED TRIAL
Source: J Rehabil Med. 2026 Jul 9;58:46254. doi: 10.2340/jrm.v58.46254 (PMC13358533; doi:10.2340/jrm.v58.46254)
Supplement: Related texts

# REPLY TO COMMENTARY ON: EFFECT OF INSPIRATORY MUSCLE TRAINING ON INSPIRATORY MUSCLE STRENGTH IN ADULTS WITH POST-COVID-19 CONDITION AND INSPIRATORY MUSCLE WEAKNESS: A RANDOMIZED CONTROLLED TRIAL

**Authors:** Anna TÖRNBERG, Anna SVENSSON-RASKH, Elisabeth RYDWIK, Alexandra HALVARSSON, Judith BRUCHFELD, Malin NYGREN-BONNIER

**Affiliations:** 1 Division of Physiotherapy, Department of Neurobiology, Care Sciences and Society, Karolinska Institutet, Huddinge; 2 Medical Unit Allied Health Professionals, Women’s Health and Allied Health Professionals Theme, Karolinska University Hospital, Stockholm; 3 Division of Infectious Diseases, Department of Medicine Solna, Karolinska Institutet, Stockholm; 4 Department of Infectious Diseases, Theme Emergency and Reparative Medicine, Karolinska University Hospital, Stockholm, Sweden

*To the Editor,*

We thank Kumar, Kaur and Chauhan for their thoughtful and constructive commentary on our trial. We fully agree that, from a rehabilitation perspective, improvements in physiological parameters are not the ultimate goal, and that reducing symptom burden, enhancing participation, and supporting meaningful engagement in daily life are of great importance. The observed discrepancy between improvements in inspiratory muscle strength and the absence of clear between-group differences in several patient-centred outcomes highlights an important area for further research. In this context, we have also conducted a qualitative study exploring participants’ experiences of the combined intervention, which we hope to publish later this year to provide additional insight into perceived benefits, barriers, and relevance in everyday life. We also concur with the emphasis on feasibility challenges. The exclusion of, and withdrawals among, individuals with more severe fatigue and post-exertional symptom exacerbation represent important clinical and methodological considerations that have not been highlighted in previous IMT trials, possibly reflecting differences in the populations studied. These findings underline the need to tailor rehabilitation approaches and to further investigate interventions that are accessible and tolerable for individuals with a higher symptom burden. Once again, we appreciate the valuable reflections in this important field.
